# System-Level Action Required for Wide-Scale Improvement in Quality of Primary Health Care: Synthesis of Feedback from an Interactive Process to Promote Dissemination and Use of Aggregated Quality of Care Data

**DOI:** 10.3389/fpubh.2016.00086

**Published:** 2016-05-04

**Authors:** Jodie Bailie, Alison Laycock, Veronica Matthews, Ross Bailie

**Affiliations:** ^1^Menzies School of Health Research, Charles Darwin University, Casuarina, NT, Australia

**Keywords:** health system and staff attributes, primary care, Aboriginal and Torres Strait Islander health, tailored interventions, quality improvement, driver diagram, aggregated quality of care data

## Abstract

**Introduction:**

There is an enduring gap between recommended practice and care that is actually delivered; and there is wide variation between primary health care (PHC) centers in delivery of care. Where aspects of care are not being done well across a range of PHC centers, this is likely due to inadequacies in the broader system. This paper aims to describe stakeholders’ perceptions of the barriers and enablers to addressing gaps in Australian Aboriginal and Torres Strait Islander chronic illness care and child health, and to identify key drivers for improvement.

**Methods:**

This paper draws on data collected as part of a large-scale continuous quality improvement project in Australian Indigenous PHC settings. We undertook a qualitative assessment of stakeholder feedback on the main barriers and enablers to addressing gaps in care for Aboriginal and Torres Strait Islander children and in chronic illness care. Themes on barriers and enablers were further analyzed to develop a “driver diagram,” an improvement tool used to locate barriers and enablers within causal pathways (as primary and secondary drivers), enabling them to be targeted by tailored interventions.

**Results:**

We identified 5 primary drivers and 11 secondary drivers of high-quality care, and associated strategies that have potential for wide-scale implementation to address barriers and enablers for improving care. Perceived barriers to addressing gaps in care included both health system and staff attributes. Primary drivers were: staff capability to deliver high-quality care; availability and use of clinical information systems and decision support tools; embedding of quality improvement processes and data-driven decision-making; appropriate and effective recruitment and retention of staff; and community capacity, engagement and mobilization for health. Suggested strategies included mechanisms for increasing clinical supervision and support, staff retention, reorientation of service delivery, use of information systems and community health literacy.

**Conclusion:**

The findings identify areas of focus for development of barrier-driven, tailored interventions to improve health outcomes. They reinforce the importance of system-level action to improve health center performance and health outcomes, and of developing strategies to address system-wide challenges that can be adapted to local contexts.

## Introduction

Despite efforts to promote best-practice clinical guideline use, adherence to guidelines remains variable between health centers and between health professionals (1–3). Interventions designed to address known barriers to care and based on evidence are more likely to produce the desired change in clinical care (4–7). Despite this knowledge, few interventions implemented are based on theory or a systematic assessment of barriers (3, 8, 9). Methods to identify barriers and to tailor interventions to address barriers need further development (4, 8, 10). In the context of major disparities in health outcomes between population groups – as between Aboriginal and Torres Strait Islander people and non-Indigenous Australians (11) – the importance of developing tailored interventions is even greater.

Large-scale improvement in the delivery of primary health care (PHC) requires change at multiple levels of the health system, not only at the local health center level (12, 13). The health system can be understood as consisting of “all organizations, people, and actions whose primary interest is to promote, restore, or maintain health” (14). Where aspects of care are not being done well across a range of PHC centers, this is likely due to inadequacies in the broader system. Ferlie and Shortell describe the health system as having four levels and argue that change is required at all four levels – those of the individual, the group or team, the overall organization, and the larger environment in which organizations are embedded – in order to improve care quality and outcomes (13). Taking a system-wide approach to continuous quality improvement (CQI) is associated with achieving large-scale improvements in health outcomes (15).

Gaps in the provision of care that may escape notice at a local level – for example, because of small numbers – can become noticeable when data are aggregated (16). In the context of limited availability of data on PHC system performance, we propose that aggregated CQI data can provide a useful source of evidence for identifying common and important gaps in care across health centers and for developing and implementing system-wide improvement efforts. A recent systematic review identified the need to seek perspectives from a range of stakeholders, such as policy and decision makers and service providers, on the health center and system attributes that lead to improved Aboriginal and Torres Strait Islander PHC outcomes (17). Reflections from a range of stakeholders in the health system can provide important insights on barriers, enablers, and strategies for improvement (17–20). The aim of this paper is, therefore, to describe stakeholders’ perceptions of the main barriers and enablers to addressing identified priority gaps in chronic illness care and child health, and to identify drivers for improvement in Aboriginal and Torres Strait Islander PHC as reflected in these stakeholder perceptions.

We have developed and implemented an active dissemination strategy – “*Engaging Stakeholders in Identifying Priority Evidence-Practice Gaps and Strategies for Improvement in PHC*” *(the ESP Project)* – that aims to promote wide-scale improvement in quality of care by applying a system-wide approach to CQI (21–23). The ESP Project is designed to engage a wide range of stakeholders in a theory-driven approach to interpret aggregated CQI data on health system performance and to reflect on barriers, enablers, and strategies for improvement (see Box 1). The rationale for the ESP Project is that involving diverse stakeholders in a phased approach of using aggregated CQI data should stimulate discussion and information sharing, and enhance ownership of the development of interventions to address system gaps. The theoretical and conceptual base for the ESP Project is described in more detail in a separate publication (21). The focus of this paper is on addressing the specific aim as described above.

Box 1 **Australian context and the ABCD National Research Partnership.**

In Australia, a high-income country with a universal health insurance scheme (Medicare), Aboriginal and Torres Strait Islander populations experience inequitable access to health care and poorer health outcomes than for non-Indigenous Australians (11). Access to PHC for Aboriginal and Torres Strait Islander people is through private general practice, government managed and Aboriginal community-controlled health services.

Strengthening PHC is critical to closing the gap in health inequalities between Aboriginal and Torres Strait Islander people and other Australians. A wide-scale CQI project, the Audit and Best Practice in Chronic Disease (ABCD) National Research Partnership (2010–2014), has employed a systems approach to improving Aboriginal and Torres Strait Islander PHC delivery (24–27). PHC centers have used evidence-based best-practice clinical audit and system assessment tools to assess and reflect on system performance, typically on an annual basis. Available CQI tools cover various aspects of PHC (e.g., chronic illness care, child health, preventive, mental, and maternal health care). Audit tools are developed through a process of expert consensus that draws upon current evidence-based care guidelines [such as CARPA (28) and Queensland Chronic Disease Guidelines (29)] (30). Over 175 Aboriginal and Torres Strait Islander PHC centers using these CQI processes voluntarily provided de-identified CQI audit data to the ABCD National Research Partnership for analysis. The strongest engagement has been from health centers in the Australian jurisdictions of the Northern Territory and Queensland.

## Materials and Methods

**Figure 1:**
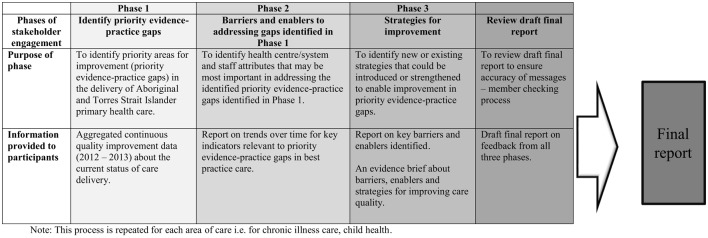
**Phases of the ESP Project for each area of care (21–23)**. Note: this process is repeated for each area of care, i.e., for chronic illness care, child health.

The theoretical basis for the phased approach for the ESP Project draws on the methods outlined by French et al. (5) to develop interventions based on evidence and identified barriers and enablers, as outlined below and in Figure 1 (21). We ran the ESP process separately for child health care and then for chronic illness care. We briefly describe each of the phases of the ESP project below by way of background. For the purpose of addressing the aim of this paper, we focus primarily on the analysis of the qualitative data derived from phase 2 of the ESP project for both child health and chronic illness care. Detailed reports on the process and findings from each of the phases of the ESP project for child health (22), chronic illness care (23), and the clinical audit tools and the audit process (25) have been published previously.

### ESP Project Phases

#### Phase 1 – Identifying the Priority Evidence-Practice Gaps

In Phase 1, a consensus-driven approach was used to identify priority evidence-practice gaps for child health and for chronic illness care. We prepared separate reports for these two areas of care, using the most recent clinical audit data (2012–2013) to describe current delivery of guideline-scheduled care across health centers, and distributed them to a range of stakeholders.

For all phases, we aimed to include individuals and organizations representing diverse roles and geographical areas, identified as having an interest and experience in Aboriginal and Torres Strait Islander health delivery, management, policy, and research. They included health practitioners (e.g., doctors, nurses, allied health professionals, Aboriginal Health Workers), managers and policy-makers at various health system levels, researchers, and staff of peak bodies and support organizations that represent the interests of community-controlled health services and Aboriginal and Torres Strait Islander communities.

Aggregated CQI data were available from 123 health centers (6,523 patient records; 90 system assessments) for chronic illness care (23) and from 94 health centers (4,011 patient records; 62 systems assessments) for child health care (22). Preliminary evidence-practice gaps were determined with the assistance of clinical experts by identifying (a) areas of clinical care that were being delivered or recorded at a relatively low level by services; (b) aspects of care where there was more general wide variation in recorded delivery of care; and (c) components of the PHC center systems that were relatively poorly developed (23). Through the survey, we asked respondents to rate the relative importance of each preliminary priority evidence-practice gap identified in the report on a scale of 1–10; and the extent to which the listed priorities resonated with their experience. Open-ended questions were used to elicit explanatory information on reasons for scores and further comments.

#### Phase 2 – Identifying the Barriers and Enablers to Addressing the Identified Evidence-Practice Gaps

In Phase 2, trend data were presented for each of the priority evidence-practice gaps identified in Phase 1 by (a) calendar years and (b) audit cycles, to show trends in variation over time and across CQI cycles. We aggregated clinical audit and systems assessment data on adherence to best practice guidelines from 160 health centers (17,879 patient records; 390 systems assessments), over the period 2005–2013 for chronic illness care; and from 132 health centers (10,405 patient records; 265 systems assessments), 2007–2013 for child health.

Through the survey, we encouraged stakeholders to reflect on the influences underlying the data trends, and on their experience in PHC, to identify barriers and enablers to improving care. The survey tool for this phase drew on international and Australian literature on health system and staff attributes (or domains) relevant to implementation of change interventions and behavior change of health care professionals (5–7, 27, 31, 32) (Table S1 in Supplementary Material lists the attributes).

Respondents were asked to rate each attribute identified according to its relative importance in improving evidence-practice gaps on a five-point Likert scale of strongly agree, agree, disagree, strongly disagree, and “do not know/cannot say.” Respondents were asked to relate their responses to providing best practice care as relevant to the priority evidence-practice gaps across the PHC system for Aboriginal and Torres Strait Islander people, rather than for any specific health center or service. Open-ended questions were also used to elicit explanatory data from stakeholders on aspects of the health system or health center environment, or staff attributes, which pose significant enablers or barriers to providing best practice care and strategies for improvement.

#### Phase 3 – Identifying the Strategies for Improvement to Address the Identified Evidence-Practice Gaps

In Phase 3, we presented a report on the barriers and enablers to addressing gaps in care identified in the previous phase. We asked respondents to comment on whether the report provided a fair reflection of the main barriers and enablers to improvement. Respondents were asked to suggest new or existing strategies to address the most common barriers and enablers.

#### Draft Final Report

In this step, we presented a draft final report on the whole process. We asked stakeholders to confirm that we had accurately reflected their feedback about strategies to address the gaps identified and whether they wished to provide additional comments that could be used to finalize the project report.

Respondents could enter data as individuals or on behalf of a group in order to encourage engagement of people who were less likely to provide individual responses. We used an iterative process to develop and refine reports through the project phases, making adjustments to content and presentation over time in response to stakeholder feedback.

Circulation lists for the ESP reports and surveys were based on networks developed over several years of the ABCD National Research Partnership. Invitations to participate were emailed by the project leader. We used a snowballing distribution technique, encouraging people to forward reports and surveys through their professional networks.

The child health ESP phases were undertaken in early 2014 and chronic illness care ESP phases in late 2014. A reminder email was sent 1 week before the closing date of surveys.

#### Data Synthesis for Identifying Common Barriers and Enablers, and Drivers for Improvement

Analysis of the difference in quantitative responses on barriers and enablers for individuals and groups showed similar response patterns. Individual and group responses were, therefore, analyzed together. Our primary interest was in the qualitative nature of responses.

For the analysis, we performed a deductive thematic analysis as described by Miles and Huberman (33). A three-step iterative process was used to identify, analyze, and describe patterns in the data, as follows. (1) The lead author (Jodie Bailie) undertook multiple readings of the interview responses. Her initial assessment of emerging themes was refined in consultation with the other authors, all of whom had been involved in the design and implementation of the ESP project and were, thus, familiar with the data. (2) Interview data were then coded systematically by the lead author, using an organizing matrix of “health system attributes” or “staff attributes” to identify common themes relating to barriers and enablers to improvement in care. (3) Three authors (Jodie Bailie, Alison Laycock, and Ross Bailie) then reviewed and conferred on the identified themes and the lead author revised the themes in light of this discussion.

We then drew on the thematic analysis to produce a “driver diagram,” an improvement tool that is used to locate barriers and enablers within causal pathways (as primary and secondary drivers), enabling them to be targeted by tailored interventions (34, 35). The process for developing the driver diagram involved the lead author further analyzing the themes on the barriers and enablers to identify system issues that could contribute to improving delivery of care (“primary drivers”) and, second, issues that could impact on these primary drivers (“secondary drivers”). Enablers and barriers were viewed as “drivers” if they represented features of the system that enabled or constrained care quality. The strategies for improvement identified by stakeholders in Phase 3 were organized according to the drivers with which they were most clearly aligned. The driver diagram then went through several iterations of review and refinement involving all authors. The refined driver diagram was subsequently presented to a group of 30 experienced practitioners and researchers working in Aboriginal and Torres Strait Islander PHC and CQI for critical feedback based on their collective knowledge and diverse roles. This feedback was incorporated and the diagram further revised to reflect this input.

#### Ethics Approval

Ethical approval for the ABCD National Research Partnership was obtained from research ethics committees in each relevant Australian jurisdiction – Human Research Ethics Committee of the Northern Territory Department of Health and Menzies School of Health Research (HREC EC00153); Central Australian Human Research Ethics Committee (HREC-12-53); New South Wales Greater Western Area Health Service Human Research Committee (HREC/11/GWAHS/23); Queensland Human Research Ethics Committee Darling Downs Health Services District (HREC/11/QTDD/47); South Australian Aboriginal Health Research Ethics Committee (04-10-319); Curtin University Human Research Ethics Committee (HR140/2008); Western Australian Country Health Services Research Ethics Committee (2011/27); Western Australia Aboriginal Health Information and Ethics Committee (111-8/05); and University of Western Australia Human Research Ethics Committee (RA/4/1/5051).

## Results

Responses for each phase and for each area of care are detailed in Table 1. For child health, there were 10–26 individual responses and 1–3 group responses in the various phases. For chronic illness care, there were 17–45 individual responses and 3–10 group responses in different phases. For chronic illness care, 10 groups who provided feedback comprised more than 20 people.

**Table 1:** **Survey respondents for child health and chronic illness care ESP project phases**.

	Phase 1 – Identifying priority evidence-practice gaps				Phase 2 – Barriers and enablers to addressing gaps in care				Phase 3 – Suggested strategies for improvement				Phase 4 – Review of draft final report			
	Child health		Chronic illness care		Child health		Chronic illness care		Child health		Chronic illness care		Child health		Chronic illness care	
	Ind.	Gr.	Ind.	Gr.	Ind.	Gr.	Ind.	Gr.	Ind.	Gr.	Ind.	Gr.	Ind.	Gr.	Ind.	Gr.
**No. of responses**	17	3	45	10	26	3	11	4	11	1	15	3	10	3	17	6

**No. of attendees per group**																

Less than 5		–		1		2		1		–		–		3		3

5–10		3		2		–		1		1		1		3

11–20		–		–		1		–		–		1		–		

More than 20		–		7		–		2		–		1		–		

**Jurisdictions of interest for respondents^a^**																

National	2		5		2		1		1		2		2		4	

New South Wales	–		1		2		1		–		–		1		–	

Queensland	11		22		6		3		4		4		4		6	

Northern Territory	6		25		16		8		5		8		5		12	

South Australia	2		5		3		1		2		4		2		6	

Western Australia	–		0		1		–		–		–		–		1	

**Rurality of population group to which responses relate^a^**																

Urban	8		15		6		4		3		9		4		8	

Regional	9		25		14		5		4		9		6		7	

Remote	15		41		24		13		10		13		11		17	

**Position types**

Nurse	4		14		5		6		3		5		4		7	

Middle Manager	1		7		1		2		2		1		2		3	

Doctor	1		11		1		4		1		5		3		3	

Public Health Physician	1		8		–		3		–		3		1		1	

Other Medical Specialist	4		4		2		–		1		1		1		1	

Senior Management/executive	–		7		–		2		–		5		1		4	

CQI facilitator	2		12		4		2		3		3		3		4	

Board member	–		2		–		–		–		1		1		–	

Policy officer	1		1		3		3		1		2		–		3	

Aboriginal Health Worker	–		5		1		1		1		1		2		2	

Research/Academic	–		9		7		2		2		2		4		2	

Other	6		12		5		1		1		4		3		1	

**Organization types**																

Community-controlled health center	1		7		4		2		6		2		6		5	

Community-controlled peak body	3		3		2		1		1		3		1		1	

Government health center	2		13		2		1		2		3		2		6	

Government health department	2		16		11		7		1		5		1		11	

Medicare local	1		2		1		–		1		–		1		–	

General practice	–		3		–		1		–		–		–		2	

University/Research organization	2		8		7		2		5		3		5		2	

Other	7		16		5		1		2		4		2		–	

*^a^Numbers may not tally with total number of respondents as respondents were able to select multiple answers*.

*Ind, individual; Gr, Group; ESP, Engaging stakeholders in identifying priority evidence-practice gaps and strategies for improvement in primary health care*.

The majority of respondents indicated that they were responding from the Northern Territory or Queensland with a remote and/or regional health-care perspective. Respondents included nurses, CQI facilitators, policy officers, doctors, researchers/academics, medical specialists, Aboriginal Health Workers, and senior and middle managers (Table 1).

Seven priority evidence-practice gaps were identified for chronic illness care and five for child health (Table 2). Common gaps across these two areas of care were related to follow-up of abnormal findings; recording of advice on risks to health; and systems for links between health centers and communities.

**Table 2:** **Priority evidence-practice gaps identified in child health care and chronic illness care (22, 23)**.

Chronic illness care	Child health
• *Follow-up of abnormal findings and review of medication*, particularly in relation to management of blood pressure, cholesterol, and glycated hemoglobin (HbA1c) *• Adherence to evidence-based current treatment guidelines* in relation to medication prescription *• Emotional wellbeing assessment and provision of support* for patients with recorded concerns *• Recording of risk factors* (including waist circumference, body mass index, and absolute cardiovascular risk assessment) and *provision of advice on risks to health* (including physical activity advice and brief interventions and referral for smokers) *• Adult vaccinations*, especially for patients with chronic kidney disease, coronary heart disease, and hypertension *• Health center systems to support high-quality care*, particularly in relation to links with community and organizational support for quality improvement systems	• Recording of all *immunizations* in child health records, and the delivery of immunizations scheduled for delivery at birth and at 2 years and older *• Monitoring and recording of key measures*, including weight, hemoglobin, and developmental milestones and follow-up action for growth faltering, anemia, chronic ear infections, developmental delay, and risks related to domestic environment, financial situation, housing, and food security *• Recording of advice or brief interventions* on child nutrition, passive smoking, infection prevention and hygiene, injury prevention, domestic/social and environmental conditions, and child development *• Recording of enquiries* made regarding use of alcohol, tobacco, and other drugs and discussion and/or advice provided on risks to health of children *• Systems for effective links* between health centers and communities and systems to support regional health planning

Common barriers to addressing care priorities for child health and chronic illness care are outlined below according to the main themes that emerged from the data. In general, the enablers were the inverse of barriers, and we have described each theme according to the balance of stakeholder perceptions as positive or negative. These main themes and illustrative quotes are presented in Table 3.

**Table 3:** **Health center/system and staff attributes that were identified as predominant barriers with example quotes**.

Health center/system attributes	Example quotes
Staffing, recruitment, and retention	*“Many barriers are a result of combination of constant changing staff and low retention of staff.” (*Nurse, Government health department, remote context, chronic illness survey, individual response)
	*“Staff employed at health* centres *are usually from emergency/acute background. There should be dedicated non-acute staff (child and family health nurses, chronic disease nurses) employed at the local level – who do not have to work on the roster for 24 hour on call – and therefore provide an uninterrupted community health* centre *to the community in partnership with Indigenous health workers which would provide much needed sustainability to program work.”* (CQI facilitator, Government health centre, remote context, chronic illness care survey, individual response)
	*“Lack of staff training, recruiting from emergency departments and just not enough permanent staff on the ground mean that brief intervention, program delivery and self-management support rarely get a look in. High staff turnover with some clinics*^a^ *having only relief staff – no permanent staff. Constantly training and orientating staff.”* (Child health survey, Group response (less than 5 people), remote)
	*“The biggest barrier is the lack of specialist child health nurses in remote health* centres *and high turnover levels of remote nurses in general so that upskilling of the remote area nurses is constant. The other biggest barrier is the lack of Aboriginal staff to work with the nurses and doctors.”* (Researcher/Academic, University or research organisation, urban, regional and remote context, child health survey, individual response)
	*“Lack of staff who actually live in the community to develop long term relationships and the lack of trained Aboriginal health workers”* (CQI facilitator, community-controlled health centre, regional and remote context, child health survey, individual response)
	*“There is a serious lack of Aboriginal health workers and allied health staff. There is also a lack of retention of these members of the workforce. It is extremely important to have Aboriginal health workers at community level with parents and carers to improve family and community practices – discussion nutrition, hygiene and when to seek care.”* (Policy officer, Government health department, remote context, child health survey, individual response)
	*“Barrier is lack of adequately trained child health nurses, and employment of generalist hospital nurses who have no idea about community, public or population health or child health.”* (Policy officer, Medicare Local, regional and remote context, child health survey, individual response)
Training and skill development	*“Not all staff are accredited to provide* immunisation*, or have the knowledge on how to document when* immunisation *is given elsewhere”* (CQI facilitator, Government health department, remote context, child health survey, individual response)
	*“Many staff go to a clinic without adequate training in the basic use of information systems and inadequate orientation to the* organisation *and the community. For some staff it is a case of they don’t know what they don’t know. Training when someone hits the ground running is difficult and in this day and age inexcusable.”* (Nurse, Government health department, remote context, chronic illness survey, individual response)
	*There are a lot of tools, training, and self-directed teaching available to staff but there is insufficient time and staffing to do all of the training constantly thrown at everyone. Due to gaps in availability of health workers and admin staff who are sufficiently trained and supported to do their jobs, the PHC facilities remain chaotic at best, particularly during periods of high turnover which continue to occur due to staff burnout/exhaustion battling in an under resourced/underappreciated and chaotic environment*. (Chronic disease survey, Group response (more than 20 people), remote context)
	*“The response to these gaps is typically to provide more packages for self-learning – eating further into front line staff time, and more management telling them they should be doing these things. There is simply insufficient number of staff to achieve every priority to the highest level.”* (Chronic disease survey, Group response (more than 20 people), remote context)
Decision support and clinical information systems	*“The electronic medical record system is extremely slow in some communities and documentation takes ages, service may have been provided more often than documented particularly in the area of advice given.”* (CQI facilitator, Government health department, remote context, child health survey, individual response)
	*“The current systems allow for creation of multiple recalls which are never able to be completed. Better system needed which provides some degree of* prioritisation *of the recalls.”* (Policy officer, Government health department, remote context, child health survey, individual response)
	*“Barrier is there are no standard guides on entering electronic data”* (Policy officer, Medicare Local, regional and remote context, child health survey, individual response)
	*“Communication between health sectors is appalling*…*. The epitome of the lack of communication are the IT services covering different health sectors and that they don’t cross reference patient information. When I would go to remote clinics (as a specialist) I would have to access four different information systems in four days – hospital, PCIS, Communicare and paper-based notes. It’s ridiculous the ehealth record has been a dismal failure, so this problem is not going away in a hurry.”* (Researcher/Academic, University or Research organisation, remote context, child health survey, individual response)
	*“The time to provide recommended care is a barrier – the electronic medical record system is extremely slow in some communities and documentation takes ages.”* (Doctor, Hospital, remote context, child health survey, individual response)
Quality improvement	*“Barrier is managers who still think CQI is extra work and not their* job” (CQI facilitator, Community-controlled peak body, remote context, child health survey, individual response)
	*“Managers who know about and understand the importance of quality improvement are also an important enabler of best practice.”* (Researcher/Academic, University or Research Organisation, regional context, child health survey, individual response)
	*“At organizational (and system) levels, there is lack of knowledge or commitment to support a culture of good clinical and information governance to ensure good documentation and assessment/management of data quality to ensure that routinely collected data are fit for clinical and quality improvement purposes.”* (Researcher/Academic, University or Research Organisation, urban and regional context, chronic illness care survey, individual response)
	*“Focused use of structured CQI methodology is dependent on the individual manager/leadership understanding and is often not consistent and well integrated into primary health care service functioning.”* (Policy officer, Community-controlled peak body, urban, regional and remote context, chronic illness survey, individual response)
Community capacity, engagement and mobilization	*“Barriers are high turnover of staff, particularly in more remote areas – relationships and networks may be made with community, local health* centres *and regional services and then need to be remade with the next staff member coming on board.”* (Researcher/Academic, University or research organisation, urban, regional and remote context, child health survey, individual response)
	*“Main barrier is community engagement and support”* (Researcher/Academic, University or research organisation, urban, regional and remote context, child health survey, individual response)
	*“Í believe an empowered and motivated community is the most important enabler for providing best practice. As long as the community demands better health care the health* centres *will improve. I have seen that health services that have a strong board to which the CEO and health workers are accountable tend to have better quality care.”* (Researcher/Academic, University or Research Organisation, regional context, child health survey, individual response)
Leadership and teamwork	*“There is a relative lack of clinical and corporate leadership to enable the implementation, training and support of evidence-based care of patients with chronic illness. There is significant lack of informatics capability among managers and clinicians to implement systems, as espoused by the chronic care model, to provide effective decision support at point of care to prompt decisions and enable evidence-based action at the clinical level. At organizational (and system) levels, there is lack of knowledge or commitment to support a culture of good clinical and information governance to ensure good documentation and assessment/management of data quality to ensure that routinely collected data are fit for clinical and quality improvement purposes such as the ABCD program”* (Researcher/Academic, University or Research Organisation, urban and regional context, chronic illness care survey, individual response)

**Staff attributes**	**Example quotes**

Intentions	*“Whilst the intention to provide best practice care is there the capacity is not, therefore it is rarely implemented both due to patient expectation – e.g. attended for acute injury not to discuss diabetes – and staffing/time issues. [There is] insufficient time to truly provide best practice care, either due to only a handful of staff trying to do all components or too many other patients who will not wait for care if it takes too long to provide patients with all aspects of best practice care in accordance with guidelines. Patients generally are either keen to be involved in their health care, come for their appointments and follow through on plans or do none of this and are captured opportunistically but as they’re not engaged it is a long drawn out process to try and provide all components of “best practice” with them.”* (Group response, + 20 doctors, remote context, chronic illness care survey)
	*“People have good intentions but are unable to do everything in the current set up with the current staffing levels”* (Chronic disease survey, Group response (more than 20 people), remote context)
	*“I truly believe that nearly every person who goes to work in remote clinics has the very best of intentions to provide the best possible care for all Indigenous children. I think that the high workload, lack of colleagues, lack of managerial support, lack of ongoing training and education and poor communication between health sectors causes issues such as “culture shock” with subsequent burn out and high staff turnover.”* (Doctor, Public hospital, remote context, child health survey, individual response)
Social influences	*“Staff often have preconceived ideas about the success of interventions with regards to Aboriginal and Torres Strait Islander populations.”* (Allied health practitioner, Government health service, remote context, child health survey, individual response)
	*“Social influences and attitude, such as new staff listening to old staff ‘it will make no difference what we tell them”; “I don’t know why we’re doing this.’”* (CQI coordinator, Government health department, remote context, child health survey, individual response)
	*“Managers feel pressured to deal with acute presentations before chronic disease. So managers need to be able to employ dedicated staff which do not deal with the day-to-day acute load.”* (CQI facilitator, Government health department, remote context, chronic illness survey, individual response)
Emotion	*“Because of the uncertainty of PHC, staff are always on edge about the future, and this transfers into care provision to the client.”* Nurse, Private practitioner, individual response, child health survey)
	*“I worry about the increasing workload for ground staff, i.e., the upcoming self-management assessments for clients, lack of health education for clients generally and staff on the ground feeling drained, incompetent and over-stretched and criticized. Many people work very, very hard and every now and then have a win but I think we need to be helping ‘on the ground’ staff a lot more than what we are currently doing.”* (Nurse, Government health department, remote context, chronic illness survey, individual response)

*^a^The term “clinic” is commonly used to refer to a health center. CQI, continuous quality improvement; PHC, primary health care*.

For both chronic illness care and child health care, respondents felt that health center and system attributes were of greater or equal importance compared to staff attributes in improving quality of care. In addition to the responses to a direct question on this issue, stakeholders’ perceptions of the relative importance of health center and system attributes were reflected in the qualitative comments. Health system barriers, such as staff shortages, were perceived to impact on staff attributes, such as emotion and intentions.

### Health Center and System Attributes

#### Staffing, Recruitment, and Retention

Respondents considered lack of systems to ensure PHC staff have support from experienced staff to be a significant barrier to improvement, especially when health centers are commonly affected by turnover and shortages of staff. Inadequate staffing levels overall, in particular a lack of Aboriginal and Torres Strait Islander-specific positions, were seen to impact on the ability to address gaps in care. Poor links between health centers and communities were viewed as a barrier to care, connected to the lack of Aboriginal and Torres Strait Islander staff to fulfill this vital role. This theme is discussed in more detail below.

Staff in remote health centers were perceived to be skilled in acute care, but not necessarily in the specific skills required for providing chronic illness or child health care. Furthermore, the demands of acute care impact on the ability of staff to provide chronic illness care. For example, staffing shortages left staff feeling unable to offer self-management support to patients and experiencing frustration because of lack of time. High staff turnover was perceived to impact negatively on the ability to implement new programs, build linkages with communities, and increase demands for health centers to offer orientation and training for new staff. Staffing issues were pervasive and accounted for the majority of respondent comments, impacting on staff morale and optimism.

#### Training and Skill Development

Skill areas in need of development included: use of clinical information systems, principles of self-management, principles of patient-centered care (especially for chronic illness care), and immunization delivery (for child health care). The limited capability of health teams to use CQI tools and processes was highlighted, with management widely perceived as being inadequately trained to support effective use of CQI tools and resources. In remote areas, nursing staff were reported to be trained in and focused on acute care rather than preventive care – this was perceived as a barrier to care in chronic illness and child health. While respondents viewed access to training, including self-directed learning packages, as generally good, high workload and time pressures on staff prevented wide uptake.

#### Decision Support and Clinical Information Systems

Respondents perceived clinical information systems as having the functionality to support provision of best practice care, and access to best practice guidelines and other decision support resources as good. They highlighted the challenge of high staff turnover and the need to constantly orient new staff to use clinical information systems effectively. There were calls for an integrated health record, accessible by multiple providers, to address challenges of providing care to populations that commonly move between communities. This population movement is particularly high in remote settings. Poor internet access for remote health centers was seen to impact on ability to use decision support guidelines and information systems, with slow systems hampering efforts to document discussions with patients.

#### Quality Improvement

Good quality improvement tools and processes were perceived as being available – particularly for chronic illness care – however, limited skill levels of managers in CQI, and in data management more generally, were seen as a barrier to supporting effective use of CQI tools and resources. CQI was reportedly not viewed as a core component of work by many staff, and was, therefore, often not prioritized. This was linked by respondents to the high turnover of staff and the need to continually train and support new staff. The identified lack of capability to use clinical information systems was seen to inhibit the effective electronic extraction and use of data for CQI purposes.

#### Community Capacity, Engagement, and Mobilization

Limitations in this area were widely regarded as barriers to improving care, with inadequate systems for increasing the expectations of community members with regard to best practice care, strengthening community leadership for health, or enhancing health literacy. Capability to build and support PHC staff to develop links to work in partnership with communities was seen as lacking, with high staff turnover impacting on the ability to build and maintain relationships between PHC staff and communities. This barrier could be linked with the strong agreement about the need for more Aboriginal and Torres Strait Islander-specific positions within health centers.

#### Leadership and Teamwork

A lack of effective leadership (often due to high staff turnover) was seen to hamper efforts to implement systems to support best practice approaches, implement CQI, and use data to inform decision-making. Inadequate use of clinical information systems by staff was seen, in turn, to hamper efforts to advance team-based approaches to care.

### Staff Attributes

In relation to assessment of staff attributes, respondents indicated that despite the good intentions of staff to provide best practice care, other episodic care arrangements and workforce shortages were seen to impact on the delivery of care. Thus, the extent to which staff attributes were impacting on quality of care appeared to be at least partly symptomatic of system-level factors. There were mixed responses in relation to the domain of emotion. Generally, respondents reported that staff enjoy their normal day-to-day activities, but they were also commonly perceived to feel unhappy, anxious, or depressed about their work.

### Drivers for Delivery of High-Quality Care

**Figure 2:**
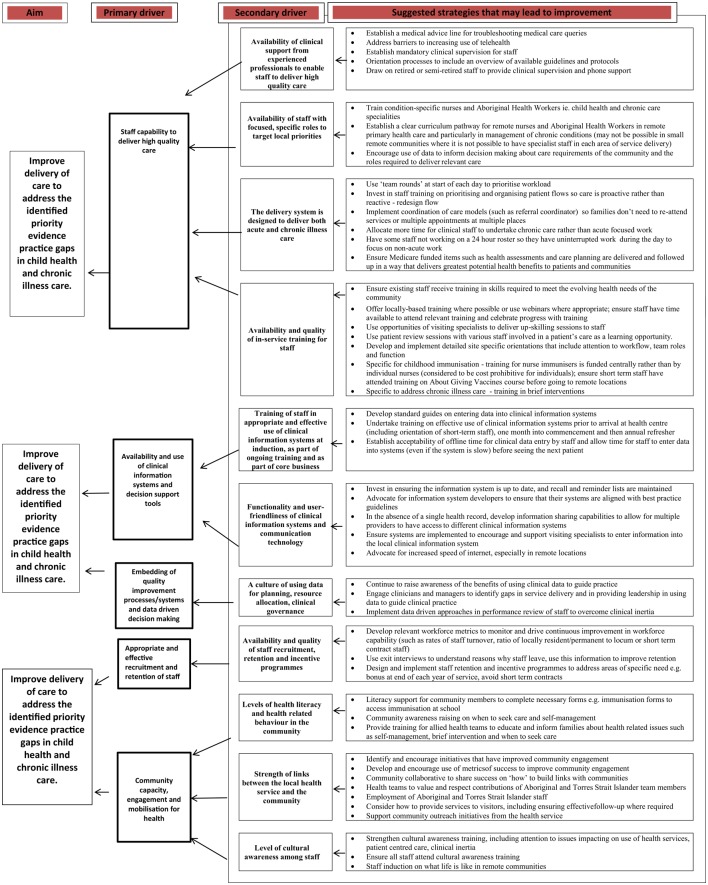
**Driver diagram showing survey responses organized as primary and secondary drivers of high-quality child health and chronic illness care and suggested strategies for addressing identified evidence-practice gaps**.

In our analysis of enablers and barriers, we identified five primary drivers that have the potential for direct impact on identified priority evidence-practice gaps in child health and chronic illness care. They were: staff capability to deliver high-quality care; availability and use of clinical information systems and decision support tools; embedding of quality improvement processes/systems and data-driven decision-making; appropriate and effective recruitment and retention of staff; and community capacity, engagement, and mobilization for health. Eleven secondary drivers were identified – these are health center system or staff attributes that have a direct impact on each of the primary drivers. There was not a one-to-one relationship between the main barriers and enablers and the primary drivers, with more than one theme being relevant to a number of the primary drivers. For example, the themes “training and skill-development,” “leadership and teamwork,” and themes related to staff attributes all have relevance to the primary driver of “staff capability to deliver high-quality care.” The drivers are presented in a Driver Diagram (Figure 2), with associated strategies. The strategies reflect actions identified by ESP respondents as having potential to influence the effect of the “drivers” in a positive direction.

## Discussion

Drawing on data from stakeholder perspectives on barriers, enablers, and strategies for improvement derived from a large-scale CQI program, we have developed a framework of 5 primary drivers and 11 secondary drivers of high-quality care. The framework offers opportunity for policy-makers to develop multi-level, barrier-driven, tailored interventions to improve delivery of care for a population that experiences marked inequities in access to health services and health outcomes.

The perception that the major barriers and enablers to improving quality of care for Aboriginal and Torres Strait Islander people relate to system design attributes, workforce, provider and patient relationships, clinical care pathways, and access is consistent with international and national literature (36). While some researchers highlight the difficulty of identifying the most important barriers or enablers for change (4), health systems strengthening frameworks have identified resources (human resources, infrastructure, financing, and knowledge); service delivery; and governance and leadership as being the core axes of the system (12). Recent work on improving Aboriginal and Torres Strait Islander PHC highlights the need to provide patient-centered care – care that is culturally safe and built on the establishment of long-term relationships (36, 37), rather than solely on implementation of evidence-based guidelines. Similarly, Van Olmen et al. (12) identify values and principles as fundamental to strengthening health systems. The feedback through the ESP process confirms that people working in the Aboriginal and Torres Strait Islander PHC sector recognize this need, and the importance of Aboriginal and Torres Strait Islander staff in meeting it, but are hampered by multi-level system constraints and practicalities of time, workload, and available workforce. The drivers and suggested strategies reflected in the driver diagram identify areas of opportunity for those developing PHC policy and interventions to develop barrier-driven, tailored interventions to improve health outcomes for Aboriginal and Torres Strait Islander people. Our hope is that the suggested strategies will spark conversations and ideas on how to address barriers to care, and that these will lead to wide-scale action for improving care.

Continuous quality improvement data from the ABCD National Research Partnership provide the most comprehensive picture available to date on the quality of PHC care received by Aboriginal and Torres Strait Islander people (26). A strength of the ESP project is that it is informed by evidence (i.e., context-specific aggregated CQI data) to identify priority evidence-practice gaps in child health and chronic illness care.

Other strengths and limitations arise from the open process used to engage stakeholders. Individuals and groups could choose to participate in any or all ESP project phases. The ESP project has relied, in part, on stakeholders sending reports to others. Thus, a limitation of the study is that it has not been possible to accurately measure reach or response rates. However, the feedback gathered through the ESP process reflects the experience and tacit knowledge from a diverse range of stakeholders, and their perceptions of the barriers, enablers, and strategies. The geographic spread of respondents, although broad, primarily represents the Northern Territory and Queensland and a remote/regional context – this may limit the generalizability of the findings. However, according to a large majority of respondents from the other jurisdictions, the priority evidence-practice gaps appear reasonably generalizable to a national level, mitigating this potential limitation. While some respondents may have had more limited experience across the PHC system than others, collectively the respondents represent perspectives from a wide range of organizations and geographic locations (as reflected in Table 1).

In recognition that barriers exist across multiple levels of the health sector, we encouraged reflection on the broader health center and system determinants of performance (27, 36). These higher system-level influences on quality of care have not been validated in the same way as questions about individual attributes based on the theoretical domains framework (5).

The collated views and ideas provide a basis for stakeholders to continue to work collaboratively across regions and jurisdictions to share knowledge and experience and develop strategies to address these known barriers and enablers. While it is widely recognized that strategies to improve the quality of care need to take account of local context, these findings reinforce the importance of multi-level action across the system to improve health center performance and Aboriginal and Torres Strait Islander health outcomes.

## Author Contributions

JB conceived the manuscript, performed the synthesis of stakeholder feedback, and drafted the manuscript. AL contributed to the synthesis of feedback and drafting of the manuscript. VM contributed to conception of ESP Project, and led the quantitative analysis of the ABCD data and the development of the ESP reports on child health and chronic illness care. RB is the leader of the ABCD National Research Partnership of which the ESP Project is an important component. He contributed to synthesis of feedback, conceptualization, and revision of the manuscript. All authors read and approved the final version of the manuscript.

## Conflict of Interest Statement

The authors declare that the research was conducted in the absence of any commercial or financial relationships that could be construed as a potential conflict of interest.

## Abbreviations

ABCD, audit and best practice for chronic disease; AHWs, Aboriginal and Torres Strait Islander health workers/practitioners; CQI, continuous quality improvement; ESP, engaging stakeholders in identifying priority evidence–practice gaps and strategies for improvement in primary health care; PHC, primary health care.
