# Supplementary material for: System-Level Action Required for Wide-Scale Improvement in Quality of Primary Health Care: Synthesis of Feedback from an Interactive Process to Promote Dissemination and Use of Aggregated Quality of Care Data
Source: Front Public Health. 2016 May 4;4:86. doi: 10.3389/fpubh.2016.00086 (PMC4854872; doi:10.3389/fpubh.2016.00086)
Supplement: Supplementary file 1 [file Table_1.docx]

*Additional file 1: Scope of health centre/system attributes and staff attributes covered by the Phase 2 surveys in the ESP Project, drawing on theoretical domains framework [5] and other literature [27, 31, 32]*

| ***Health centre and system attributes*** | |
| --- | --- |
| Financing and resources | Within the socio-political context, there is sufficient financial support (e.g. from local/regional health authorities, government) to support best practice in chronic illness care / child health as relevant to the priority evidence-practice gaps |
| Facilities and equipment | PHC facilities are generally of adequate size, design, and condition |
|  | PHC facilities generally have adequate equipment |
| Staffing/workforce | PHC centres generally have adequate numbers of staff |
|  | PHC centres generally have appropriate types of each of the following categories of staff: |
|  | Nurses |
|  | Aboriginal or Torres Strait Islander Health Workers |
|  | Doctors (GPs) |
|  | Medical Specialists |
|  | Allied health workers |
|  | Administrative staff |
| Staff/workforce support, recruitment and retention | There are good systems in place to ensure PHC staff have support from experienced staff, especially when health centres are affected by turnover of staff and staff shortages |
|  | There are good systems in place to recruit, retain and support each of the following categories of staff (full time or visiting) as integral members of PHC teams: |
|  | Nurses |
|  | Aboriginal or Torres Strait Islander Health Workers |
|  | Doctors (GPs) |
|  | Medical Specialists |
|  | Allied health workers |
|  | Administrative staff |
| Teamwork | PHC staff function effectively in teams |
|  | PHC staff are generally clear about their roles in relation to other members of the PHC team |
|  | PHC staff are trained to work effectively in teams |
| In service training and development | There are good systems in place to support staff development, including the development of knowledge and skills required |
|  | There are good systems in place to support inter-organisational and intra-organisational learning |
| Self-management | Staff are well trained in the principles of client self-management as relevant to chronic illness care / child health |
|  | There are good self-management resources that are relevant to chronic illness care/child health available to PHC staff |
| Patient centred care | There are good systems in place to support all members of PHC teams in understanding the needs and aspirations of people living in Aboriginal and Torres Strait Islander communities for the purpose of providing best practice care in chronic illness care/ child health |
|  | There are good systems in place to support all members of PHC teams to provide care that is respectful of and responsive to individual patient preferences, needs, and values, and ensuring that patient values guide all clinical decisions |
|  | There are good systems in place to train all members of PHC teams in providing patient-centered care for people living in Aboriginal and Torres Strait Islander communities |
| Population health | There are good systems in place to ensure PHC teams have a clear understanding of the size, diversity and other key features of their service populations and to apply this knowledge |
|  | There are good systems in place to ensure PHC teams are able to apply the principles of population health |
|  | There are good systems in place to ensure PHC teams are well trained in the principles of population health |
| Decision support | Best practice guidelines and other decision support resources are available to PHC staff |
|  | PHC staff are adequately trained to use the available best practice guidelines and other decision support resources |
| Clinical information systems and communication technology | The clinical information systems and communication technology in place have the functionality to support provision of best practice care |
|  | PHC staff are trained and effectively supported to use clinical information systems and communication technology for supporting and providing best practice |
| Quality Improvement | There are good quality improvement tools available in health centres for supporting and improving delivery of best practice care |
|  | PHC staff are adequately trained to use quality improvement tools and resources for supporting and improving delivery of best practice care |
|  | Managers are adequately trained to support effective use of quality improvement tools and resources for monitoring and enhancing delivery of best practice care |
|  | There is good local ownership by PHC staff of CQI data and CQI processes for supporting and improving delivery of best practice care |
|  | Managers provide clear and appropriate support for effective use of quality improvement tools and resources by PHC staff for monitoring and enhancing delivery of best practice care |
|  | PHC staff generally believe that CQI data and CQI processes can be used for supporting and improving delivery of best practice |
| Community capacity, engagement, mobilisation | There are good systems in place to increase the expectation of community members with regard to best practice care |
|  | There are good systems in place to strengthen community leadership for quality with regard to best practice |
|  | There are good systems in place to enhance the health literacy of community members with regard to best practice care |
|  | There are good systems in place to build the capability and to support PHC staff to develop effective links to work in partnership with the communities they serve in providing best practice care. |
| Leadership and Management | There is good clinical and management leadership at the regional, state, national level for supporting and providing best practice care |
|  | There are good networks and regional coordination between parties involved in supporting and providing best practice care |
|  | Managers actively support the development of partnerships across the health sector for the purpose of enhancing delivery of best practice care |
| **Staff attributes** | |
| Knowledge | PHC staff know the content and objectives of best practice care |
|  | PHC staff are aware of how to provide best practice care for Aboriginal and Torres Strait Islander people with a chronic illness / child health |
| Skills | PHC staff have the skills to provide best practice care for Aboriginal and Torres Strait Islander people with a chronic illness / child health |
| Social/professional identity | PHC staff recognise that it is their professional responsibility to provide best practice care for Aboriginal and Torres Strait Islander people with a chronic illness / child health |
| Beliefs and capabilities | PHC staff are confident in their ability to provide best practice care for Aboriginal and Torres Strait Islander people |
| Optimism | With regard to providing best practice care for Aboriginal and Torres Strait Islander people with a chronic illness/ child health, PHC staff are optimistic about the future |
| Beliefs about consequences | PHC staff believe that if they provide best practice care for Aboriginal and Torres Strait Islander people with a chronic illness/ child health, it will have benefits for the health of Aboriginal and Torres Strait Islander people more generally at a population level |
| Intentions | For every 10 Aboriginal and Torres Strait Islander people attending PHC services, for how many would the PHC staff (on average) intend to provide best practice care? (scale of 1 – 10) |
|  | Intention of PHC staff to provide best practice care every day to Aboriginal and Torres Strait Islander people attending their services? (Mostly strong, always strong, quite strong, not strong, don’t know/can’t say) |
| Memory, attention and decision processes | PHC staff believe that when they need to concentrate to provide best practice care for Aboriginal and Torres Strait Islander people, they have no trouble focusing their attention |
|  | How often do PHC staff remember to provide best practice care for Aboriginal and Torres Strait Islander people? (Always, often, occasionally, don’t know/can’t say) |
| Social influences | PHC staff believe that if they provide best practice care for Aboriginal and Torres Strait Islander people it will have disadvantages for their relationships with Aboriginal and Torres Strait Islander people. |
| Emotion | Do PHC staff who currently provide care for Aboriginal and Torres Strait Islander people feel unhappy, anxious or depressed about their work? (Always, often, occasionally, don’t know/can’t say) |
|  | Are PHC staff who provide care for Aboriginal and Torres Strait Islander people currently able to enjoy their normal day-to-day work activities? (Always, often, occasionally, don’t know/can’t say) |

PHC - primary health care; CQI – continuous quality improvement
